# Supplementary material for: Long-term optical imaging of the spinal cord in awake, behaving animals
Source: bioRxiv. 2023 May 24:2023.05.22.541477. Preprint. [Version 1] doi: 10.1101/2023.05.22.541477 (PMC10245895; doi:10.1101/2023.05.22.541477)
Supplement: Supplement 7 [file NIHPP2023.05.22.541477v1-supplement-7.pdf]

bioRxiv preprint doi: https://doi.org/10.1101/2023.05.22.541477; this version posted May 24, 2023. The copyright holder for this preprint (which was not certified by peer review) is the author/funder, who has granted bioRxiv a license to display the preprint in perpetuity. It is made available under aCC-BY 4.0 International license.

SUPPLEMENTARY VIDEOS

Supplementary Video 1: Normal behavior of mice with implanted spinal cord chamber (Fig. 1j)
Mice in homecage 40 days (green tape) and 50 days (yellow tape) after chamber implant are very active.

Supplementary Video 2: Chamber implant (Fig. 1d)
Step-by-step guide for attachment of the spinal chamber to the vertebral column; playback speed for each surgical step indicated on top left.

Supplementary Video 3: Laminectomy (Fig. 1d)
Step-by-step guide for laminectomy and placement of PRECLUDE to inhibit fibrosis; playback speed for each surgical step indicated on top left.

Supplementary Video 4: Window placement (Fig. 1d)
Step-by-step guide for PRECLUDE removal, Teflon AF application, and placement of circular glass coverslip window; playback speed for each surgical step indicated on top left.

Supplementary Video 5: microCT sagittal slice (Fig. 1g-i)
Sagittal slice through mouse with BioMed Clear spinal chamber implanted at T12-L1 and following laminectomy.

Supplementary Video 6: microCT 3D reconstruction (Fig. 1i)
3D rendering of microCT, post-laminectomy (T13), showing sequential removal of soft tissue (brown), spinal implant chamber (white), and glass window (red), leaving just the bone (gray).

Supplementary Video 7: Open field tracking of mice with spinal implant (Fig. 1l-n)
Open field tracking of behavior post-laminectomy for DeepLabCut network trained (top row) and network naive (bottom row) movies.

Supplementary Video 8: Large shift motion correction using deep learning, control point registration, and rigid correction (Fig. 2c-f)
Large rostrocaudal shift motion correction using LD-MCM compared to TurboReg and NorMCorre during spinal cord projection neuron recording in a Phox2a-Cre; Ai162 (GCaMP6s); Ai9 (tdTomato) mouse. Dots indicate features tracked using DeepLabCut in each movie; black bars in movies indicate sections of no usable data.

Supplementary Video 9: Deformation correction using displacement fields (Fig. 2h-k)
Non-rigid motion correction using NR-MCM compared to NoRMCorre and TurboReg, during spinal cord projection neuron recording in a Phox2a-Cre; Ai162 (GCaMP6s); Ai9 (tdTomato) mouse.

Supplementary Video 10: Long-term Thy1-GFP spinal cord imaging, for over a year (Fig. 3c)
Long-term imaging of a Thy1-GFP mouse after CS-MCM motion correction and with DeepLabCut tracking of vasculature features (colored dots).

Supplementary Video 11: Time-lapse spinal cord expression of GFP and tdTomato after retro-orbital injection (Fig. 3i-m)
Time-lapse imaging of increasing GFP and tdTomato expression before and after retro-orbital injection of AAV-PHP.eB-GFP and AAV-PHP.S-tdTomato.

Supplementary Video 12: Rapid changes in fluorescence and vasculature upon isoflurane induction (Fig. 3p)
Recording of change in vasculature diameter and Thy1-GFP fluorescence during a single session as the mouse enters and exits general anesthesia (2% isoflurane). Time on the bottom left is hours:minutes:seconds.

bioRxiv preprint doi: https://doi.org/10.1101/2023.05.22.541477; this version posted May 24, 2023. The copyright holder for this preprint (which was not certified by peer review) is the author/funder, who has granted bioRxiv a license to display the preprint in perpetuity. It is made available under aCC-BY 4.0 International license.

Supplementary Video 13: Somatotopic mapping of spinal cord activity in response to caudal body stimulation (Fig. 4i-k)
Bulk imaging of GCaMP6s activity after stimulation of indicated body parts from Ai162 mouse injected with AAV2retro-hSyn-Cre into the spinal cord.

Supplementary Video 14: Imaging spinal cord projection neurons in response to various stimuli in the awake, behaving mouse (Fig. 5d-g)
Awake spinal cord imaging session in response to noxious thermal and mechanical stimuli. Video shows spinal cord projection neuron activity (Phox2a-Cre; Ai162), behavior from multiple camera angles (colored squares indicate DLC tracking points), locomotion (white, rotary encoder), head movement (cyan), and stimulus application (red).

Supplementary Video 15: Cross-day imaging of the same area of the dorsal horn in anesthetized and awake states, over a month (Fig. 5i)
Recording of spinal cord projection neuron activity across 20 days, before and after applying noxious heat to the right hindpaw (red bar on left side of movie). Days 7-8 are under isoflurane anesthesia.

Supplementary Video 16: Multiplane imaging of monocytes in a CX3CR1-EYFP mouse (Fig. 6d)
Two-photon imaging of monocytes (CX3CR1-EYFP mouse under 2% isoflurane) showing multiple planes from the spinal cord parenchyma to the meninges during a ~15-min session.

Supplementary Video 17: Cross-day timelapse shows increased nerve injury-induced CX3CR1-mediated GFP microglia expression (Fig. 6g)
Time-lapse imaging for months of microglia dynamics before and after nerve injury (left hindpaw, SNI model). Red regions indicate areas of greater fluorescence.

bioRxiv preprint doi: https://doi.org/10.1101/2023.05.22.541477; this version posted May 24, 2023. The copyright holder for this preprint (which was not certified by peer review) is the author/funder, who has granted bioRxiv a license to display the preprint in perpetuity. It is made available under aCC-BY 4.0 International license.

SUPPLEMENTARY TEXT

Surgical considerations for chamber implant and assembly

The side bar position will vary slightly for each mouse, so the freedom of movement of the side bars greatly facilitates the positioning of the chamber during surgery. To adjust the width of the metal chamber, instead of using small metal screws that are difficult to handle, we use Super Glue to attach the metal parts. Metal surfaces covered with Super Glue are then clamped together setting a strong bond, which obviates the need for set screws. Surgifoam® is used in the first procedure for hemostasis and protection of the intervertebral tissue, before Kwik-Sil. To keep the tissue hydrated, saline-soaked Surgifoam pieces are placed in intervertebral areas and in rostral and caudal areas of open tissue, until the bars are in place. After the bars are in position, the Surgifoam is removed and the space is briefly dried with a sponge. Small drops of Kwik-sil are then applied to cover the intervertebral space; these sit at room temperature until cured. The Kwik-sil mask is maintained throughout cementing steps. Critically, the mask protects intervertebral space from the dentin activator and liquid cement, which are applied in bulk to free-flow around the mask. An unprotected intervertebral space is vulnerable to chemical and mechanical injury, which will not only create a lot of bleeding but also lead to permanent damage to the spinal meninges and spinal cord.

The needles that are inserted perpendicular to the spinal column, through the dorsal spinous processes (DSP), are a key step and allow the components to be constructed around the vertebrae. We use a 33G needle (Accuderm, NP335) with a beveled edge and a bore size diameter of 0.2 mm. Place the needle on a 1mL syringe and rotate the cutting edge to facilitate driving it through the DSP bone. When the needle is through, snip the needle at the base of the shaft to have equal lengths of the needle on each side.

Extended Data Fig. 2a-i illustrates the cross-sectional anatomy of a vertebra and the location of the dorsal spinous process, facet joints, and lamina, and how the side bars are positioned relative to these vertebral landmarks. Note that only the facet joints surrounding the middle vertebrae (T13) are exposed during surgery. Before cement application, it is important to debride any tissue above the vertebral bone as this tissue could come into contact with the cement. To further improve the bone cement (PMMA) connection to the bone, a pretreatment with a dentin activator (included in Metabond kit) is recommended. Of course, any cement placed

bioRxiv preprint doi: https://doi.org/10.1101/2023.05.22.541477; this version posted May 24, 2023. The copyright holder for this preprint (which was not certified by peer review) is the author/funder, who has granted bioRxiv a license to display the preprint in perpetuity. It is made available under aCC-BY 4.0 International license.

on soft tissue or ill-prepared bone will not hold, as can happen when blood debris sticks to the bone. Before installing the stabilizing plate, gently press down the middle vertebrae and carefully lower both side bars, so that the alignment between vertebrae is as flat as possible before fixation.

Post-operative recovery

Robust recovery

The animals are fully mobile after surgery. At 24 hrs, the animals are somewhat limited in their speed of movement but are alert, ambulate, and groom. By 48 hrs, the animals spontaneously rear and show normal movements.

Implant location considerations

Placing side bars under the facet joints surrounding the middle vertebra of interest (along with the dorsal spinous process needles on the flanking vertebrae) can theoretically be attempted at any triplet of vertebrae along the spine. For instance, we were able to successfully image the L6/S1 spinal cord by a T13-L2 side bar placement and L1 laminectomy (**Fig. 4i-k**). However, L1 animals commonly presented with severe urinary retention and succumbed 48-72 hours after surgery. We have not explored whether other rostrocaudal positions are possible. Post-op excretion of urine through manual compression at 24 hours, under anesthesia, was necessary for survival in cases associated with L1 surgery. In some cases, there were post-op complications with a slow-release form of Buprenorphine, namely, Ethiqa.

Cutaneous self-injury

Occasionally chemical or mechanical intervertebral injury during surgery can produce a focal, but treatable, problem. The animals will show focal skin lesions due to self-inflicted biting. For the T13-centered implant, which overlies the L4/L5 spinal cord segments, biting occurred in the L4/L5 dermatome, including the hindpaw. Attention to the intraoperative, intervertebral mask using Kwik-sil, will prevent the potential toxicity of liquid dentin activator (ferric chloride) or uncured cement (PMMA).

Mechanism of fibrosis inhibition with fluoropolymers

In previous spinal cord optical imaging methods (Farrar et al., 2012), post-laminectomy fibrosis was abated by room-temperature vulcanizing (RTV) silicone adhesive, namely, Kwik-sil, applied directly over the meninges. However, we found that the dura becomes re-established under a bare Kwik-Sil or a precured Polydimethylsiloxane (PDMS) layer. Also, we commonly observed that, within a month, fibrosis that blocks the field of view emerges under the silicone layer. Therefore, we adopted two fluoropolymer materials, GORE® PRECLUDE® Pericardial Membrane, and Teflon™ AF. To skip the intermediate PRECLUDE® membrane step entirely, we attempted untreated Teflon AF placement immediately after laminectomy, but this approach failed. PRECLUDE®'s greater effectiveness at inhibiting fibrosis after laminectomy may be due to its microporous texture, which may accelerate the mechanical adhesion of meningeal cells. Teflon AF has a remarkably low surface energy and thus a molecular coating of hydrophilic units may be needed for adhesion, similar to another fluoropolymer, CYTOP, and its successful interface with the brain meninges after PEO-coating (Takahashi et al., 2020). Indeed, increasing the hydrophilicity of Teflon AF, by molecular etching, specifically an oxygen plasma coating (Harrick Expanded plasma cleaner PDC-001, 3 minutes at 55W), demonstrated improved compatibility over untreated Teflon AF in the post-laminectomy period. However, to establish a strategy that does not require sophisticated material pretreatment and is likely more accessible to the field, we applied the microporous PRECLUDE® membrane immediately after laminectomy and then swapped for the Teflon AF membrane in a second operation.

The mechanism of the fibrosis-inhibiting properties of ePTFE, in the form of PRECLUDE®, or Teflon AF is unresolved. In some cases, we observed dramatic evidence of transient meningeal neo-vascularization after placing Teflon AF (Extended Data Fig. 8). What is clear is that for months to years, surgically implanted PRECLUDE® Pericardial membranes develop few to no adhesions, allowing the material's quick removal and an overall decrease in time of cardiovascular surgery at reoperation (Loebe, 1993; Zehr, 1993). There are also reports of ePTFE's effectiveness in preventing postoperative peridural fibrosis (Cemil, 2009), which has major implications in combating Failed-back syndrome. Again, the outstanding hydrophobic, low surface energy and no nonspecific absorption properties of Teflon contribute to its chronic biocompatibility without integrating

bioRxiv preprint doi: https://doi.org/10.1101/2023.05.22.541477; this version posted May 24, 2023. The copyright holder for this preprint (which was not certified by peer review) is the author/funder, who has granted bioRxiv a license to display the preprint in perpetuity. It is made available under aCC-BY 4.0 International license.

physically with the tissue. The overarching theory is that Teflon is an inert physical material that can prevent mechanical contact between meningeal layers and connective tissue, for an extended time, and thus would allow independent healing of the meningeal surface (Ahmad, 2020). In this mechanism, ePTFE is an effective barrier agent above the spinal cord. Thus, dural regeneration and fibrosis are marginalized to locations that are not problematic for imaging. This mechanism has also been suggested to enable cranial imaging studies (Shtoyerman et al., 2000; Chen et al. 2002). Other materials, namely polyethylene terephthalate (PET) film, used in brain imaging protocols may play a similar role as Teflon AF, though remain untested as agents that could preserve spinal visibility (Ghanbari et al., 2019). In future studies, it is possible that reducing the thickness of the Teflon AF to a nanosheet would adhere more tightly to the spinal meninges, than did the thicker membranes used in this study. Though nanosheet handling is difficult, fluoropolymer nanosheets of 130 nm thickness, namely CYTOP, have proven sufficient for long-term cranial imaging (Takahashi et al., 2020). Interestingly, in future approaches, as nanosheets are penetrable, it may be possible to permit electrode manipulation, optical recording, and chronic micro-injections in a stable state of fibrosis inhibition.

Equipment and materials

Customized surgical table

To construct the spinal cord surgical station, two Thor labs 3-axis manipulators equipped with clamps were installed on an MB618 plate. In the middle of the clamps, two MB412 plates created slanted armrests for a customizable, 3D-printed surgical bed, following previous designs (Farrar et al., 2012). Additional components of gas delivery (Posi-vac, Patterson Scientific), active waste gas scavenging (EVAC 2, Patterson Scientific), vacuum aspirator (in-house vacuum flask), and isothermic bed (FHC) were integrated into the custom surgical set-up. For details see: (**Extended Data Fig. 1c**) and the GitHub containing CAD files of the individual 3D printed components.

bioRxiv preprint doi: https://doi.org/10.1101/2023.05.22.541477; this version posted May 24, 2023. The copyright holder for this preprint (which was not certified by peer review) is the author/funder, who has granted bioRxiv a license to display the preprint in perpetuity. It is made available under aCC-BY 4.0 International license.

Step-by-step surgical procedure

The following step-by-step surgical procedures are complemented by diagrams in **Supplemental Fig. 1-3** and **Supplementary Videos 2-4**.

Chamber implant (see Supplemental Fig. 1 and Video 2)

Step	Description of surgical steps (chamber implant)
-	Pre-op checklist: Sterilize tools, sterilize implantable components (side bars & stabilizing plate), wet hemostatic sponge (Surgifoam) with saline, prepare 0.5% lidocaine solution, weigh mouse and prepare NSAID analgesic, induce anesthesia, stabilize body temperature and respiratory rate, apply eye ointment, remove hair over the lumbar enlargement, disinfect the skin, administer NSAID analgesic.
1	Position the animal on the surgical table so that the lumbar enlargement is in line with the side posts *Drape the surgical field to improve sterile technique.
2	Confirm loss of the toe pinch reflex, then incise and open ~1.0 cm of skin along the midline overlying the lumbar enlargement. *Apply intra-incisional lidocaine.
3	Locate the T12, T13, and L1 vertebrae.
4	Using sharp, pointed microscissors, penetrate fascia and paraspinal muscles lateral to each dorsal spinous process (DSP) of T12, T13, and L1 vertebrae, and elevate muscles from laminae by blunt dissection. *Apply Surgifoam if there is bleeding.
5	Lift and cut away the loose paraspinal muscle lateral to the T12, T13, and L1 DSPs *Do not cut into the intervertebral muscle. *To maintain tissue hydration until Step 11, place saline-soaked Surgifoam over the intervertebral muscle.
6	Sever the thick tendons connected to the articular processes of T13. *Optional: To more easily reveal these tendons, open a retractor under the muscles overlying the T13 lamina.
7	Wipe or scrape clean the bone surfaces of all laminae & DSPs. *If necessary, remove any remaining muscle adhered to the laminae.
8	Insert a 33G needle through the T12 DSP and snip the base of the needle. Repeat at L1 DSP.
9	Clamp the side bars to the side posts and slide each into the surgical field. Lift the needles until they sit over the top of the side bars.
10	Use a micro spatula to progressively tuck the exposed muscle and skin slowly under the side bars while advancing the side bars towards and against the vertebral column. *Adjust the vertebral column by pressing down on T13 and lowering the side bars before the side bars contact the vertebrae. *To create a flat base for stabilizing plate attachment (Step 16), level the side bars with each other.
11	Remove all saline-soaked sponges, blot the field dry, and immediately make a discrete Kwik-sil mask to cover all intervertebral spaces.
12	Affix needles to the side bars with Superglue contacts.
13	As the glue dries, suture the incised skin together rostral and caudal to the side bars.
14	Adjacent to the suture, seal the rostral and caudal tissue border with a moderate amount of Vetbond.
15	To free the side bar and needle construction, release the side post clamps.

bioRxiv preprint doi: https://doi.org/10.1101/2023.05.22.541477; this version posted May 24, 2023. The copyright holder for this preprint (which was not certified by peer review) is the author/funder, who has granted bioRxiv a license to display the preprint in perpetuity. It is made available under aCC-BY 4.0 International license.

Step	Description of surgical steps (chamber implant)
16	Apply Superglue onto each side bar handle and then place the stabilizing plate over top and manually squeeze to glue together and then re-engage the side clamps. *Add additional Superglue at the inside notches of the stabilizing plate when clamped.
17	To maximize the area of bone cement binding, scrape the exposed laminar bone of each vertebra, without disrupting the intervertebral mask.
18	To further prepare the bone for bone cement binding, apply dentin activator gel to bone surfaces for 15 seconds; then rinse off with saline and blot dry.
19	Generously apply free-flowing cement to the field, coating the prepared bone, DSP needles, and side bar edges.
20	To access the T13 lamina for future laminectomy, immediately clear any cement from the T13 lamina before it sets.
21	Finally, to protect the exposed lamina until laminectomy, coat the T13 lamina and surrounding cement with Kwik-sil.
-	Post-op checklist: Provide heat, monitor recovery, and administer opioid analgesic.

Laminectomy (see Supplemental Fig. 2 and Video 3)

Step	Description of surgical steps (laminectomy)
-	Pre-op checklist: Weigh the mouse and prepare NSAID analgesic, induce anesthesia, stabilize body temperature and respiratory rate, and apply eye ointment.
1	Remove the Kwik-sil coat over the T13 lamina. *If necessary, reduce cement at the edges of the T13 lamina with a microdrill burr.
2	Cut through the lamina as laterally as possible, on both sides.
3	Transect the intervertebral ligaments to free the lamina and then remove the lamina *Take care to separate any attachments to the lamina before pulling away.
4	Immediately add saline-soaked hemostatic sponge over the spinal cord to maintain hydration and stop any bleeding.
5	Separate the dural layer and retract laterally to expose the leptomeninges (arachnoid and pia mater) and replace the hemostatic sponge. *Widen the laminectomy, if necessary, to expose the characteristic grayish hue of the dorsal horn.
6	Shape a single piece of the PRECLUDE membrane to the size of the exposed spinal cord.
7	Remove Surgifoam and place the PRECLUDE membrane directly over the spinal cord in contact with the leptomeninges.
8	Place a small amount of saline-soaked Surgifoam around the border of the Preclude membrane (this helps limit ingrowth).
9	Blot dry the Surgifoam and apply Kwik-sil to close off the entire field and hold the Preclude membrane in place until window placement.

bioRxiv preprint doi: https://doi.org/10.1101/2023.05.22.541477; this version posted May 24, 2023. The copyright holder for this preprint (which was not certified by peer review) is the author/funder, who has granted bioRxiv a license to display the preprint in perpetuity. It is made available under aCC-BY 4.0 International license.

Step	Description of surgical steps (laminectomy)
-	Post-op checklist: Provide heat, monitor recovery, and administer NSAID analgesics.

Window placement (see Supplemental Fig. 3 and Video 4)

Step	Description of surgical steps (window placement)
-	Pre-op checklist: Weigh the mouse and prepare NSAID analgesic, induce anesthesia, stabilize body temperature and respiratory rate, and apply eye ointment.
1	Remove the Kwik-sil coat and PRECLUDE membrane to expose the spinal cord.
2	Immediately add saline-soaked hemostatic sponge over the spinal cord to maintain hydration and stop any bleeding.
3	Shape a single piece of the Teflon AF membrane to the size of the exposed spinal cord and place it directly over the spinal leptomeninges.
4	Add a small amount of Kwik-sil to flow over the Teflon AF & laminectomy area.
5	Immediately set a 3.0 mm circular coverslip *Press down on the coverslip to squeeze out the excess Kwik-Sil adhesive.
6	Allow Kwik-sil adhesive to harden for 10 min.
7	Permanently set the coverslip with bone cement or Norland optical adhesive *Completely cover the edges of the Kwik-sil layer to seal the preparation.
-	Post-op checklist: Provide heat, monitor recovery, and administer NSAID analgesics.

bioRxiv preprint doi: https://doi.org/10.1101/2023.05.22.541477; this version posted May 24, 2023. The copyright holder for this preprint (which was not certified by peer review) is the author/funder, who has granted bioRxiv a license to display the preprint in perpetuity. It is made available under aCC-BY 4.0 International license.

SUPPLEMENTARY FIGURES

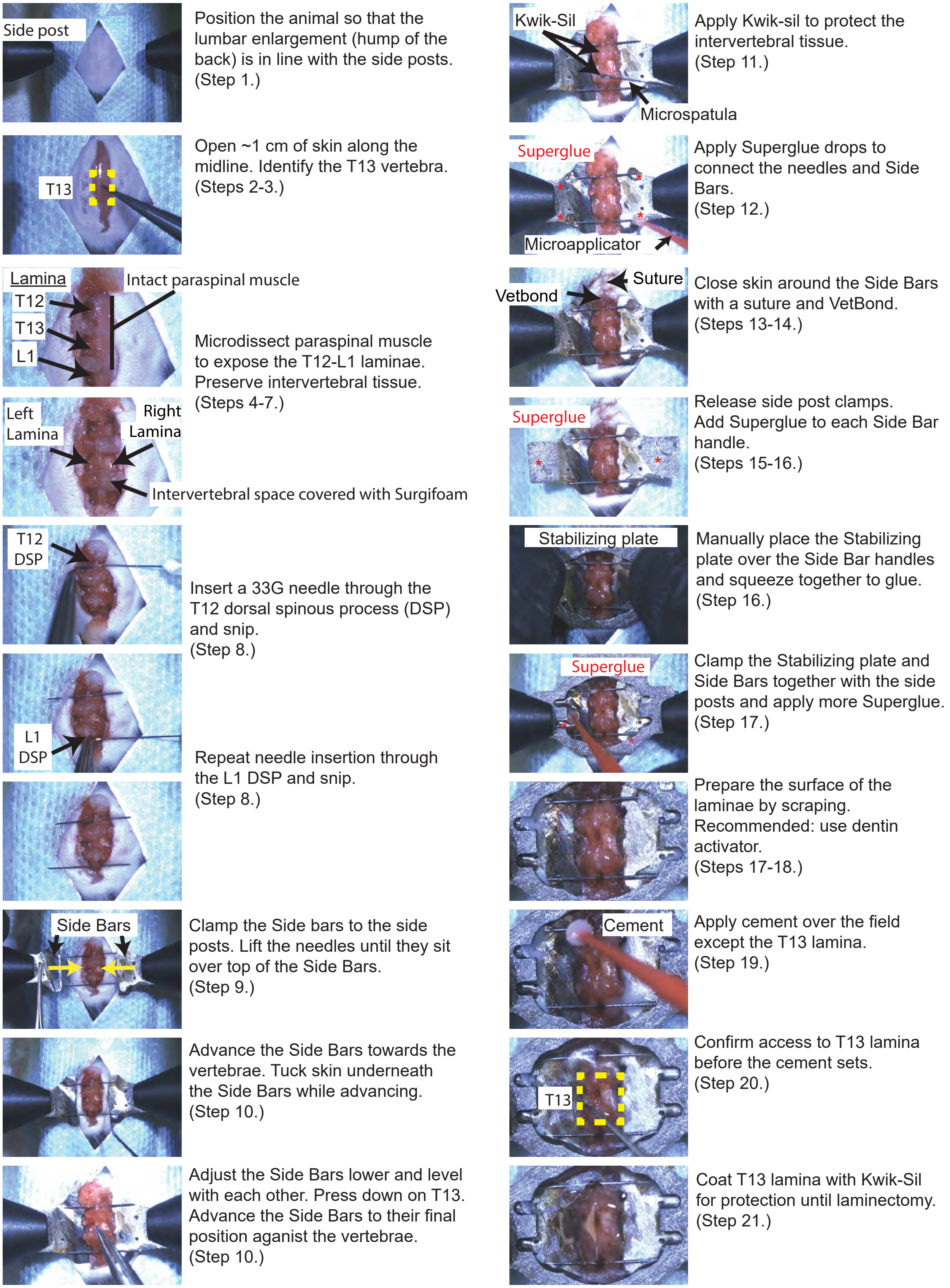

Supplementary Fig. 1 | Key frames of the first procedure - Chamber implantation
Three metal pieces are implanted paraspinally, in 21 steps.

bioRxiv preprint doi: https://doi.org/10.1101/2023.05.22.541477; this version posted May 24, 2023. The copyright holder for this preprint (which was not certified by peer review) is the author/funder, who has granted bioRxiv a license to display the preprint in perpetuity. It is made available under aCC-BY 4.0 International license.

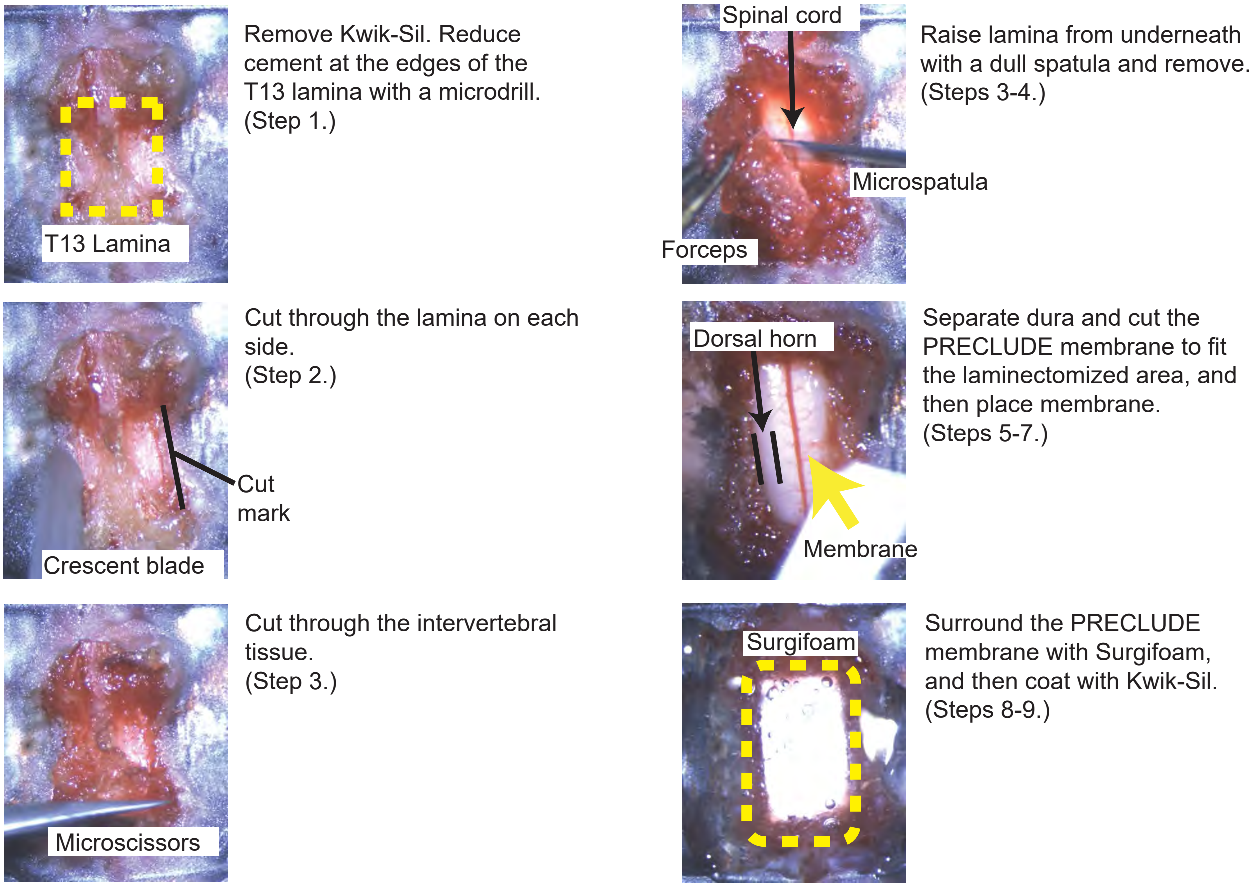

Supplementary Fig. 2 | Key frames of the second procedure - Laminectomy
1 week or more after the bar-implantation, a laminectomy is performed in 9 steps.

bioRxiv preprint doi: https://doi.org/10.1101/2023.05.22.541477; this version posted May 24, 2023. The copyright holder for this preprint (which was not certified by peer review) is the author/funder, who has granted bioRxiv a license to display the preprint in perpetuity. It is made available under aCC-BY 4.0 International license.

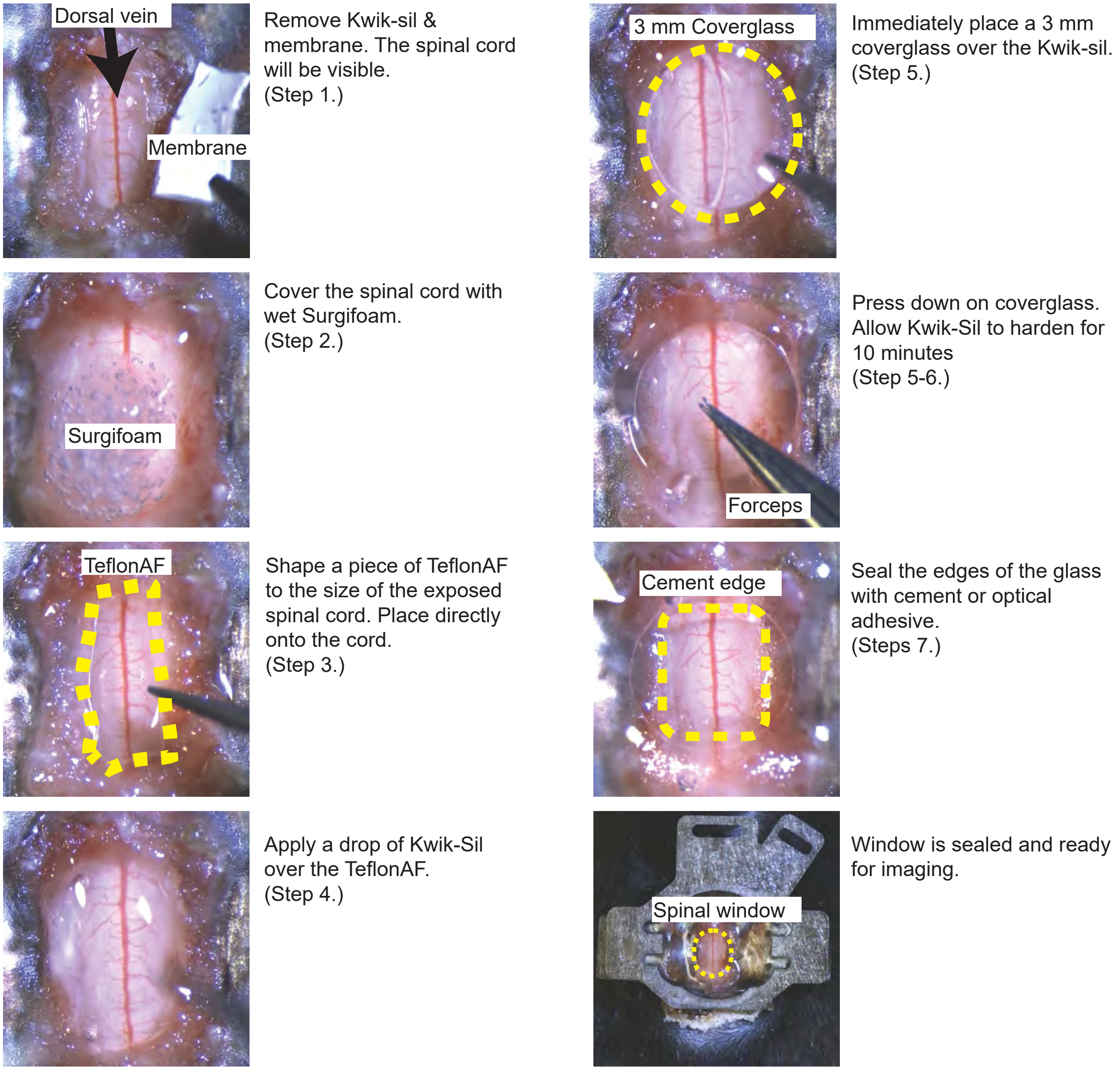

Supplementary Fig. 3 | Key frames of the third procedure - Spinal cord window placement
A glass window is installed by following these 7 steps.

bioRxiv preprint doi: https://doi.org/10.1101/2023.05.22.541477; this version posted May 24, 2023. The copyright holder for this preprint (which was not certified by peer review) is the author/funder, who has granted bioRxiv a license to display the preprint in perpetuity. It is made available under aCC-BY 4.0 International license.

SUPPLEMENTARY TABLES

Supplementary Table 1 | Materials used for in vivo imaging of the mouse spinal cord

Item	Vendor	Product No.	Website	Experiment/Note
**Analgesics**				
Ethiqa XR	Fidelis Animal Health, Inc.	86084-100-30	https://ethiqaxr.com/	Surgery (general use)
Lidocaine	Hospira Inc	0409-2066-10	https://www.pfizermedicalinformation.com/en-us/lidocaine/principal-display	Surgery (general use)
Carprofen	Zoetis Inc.	54771-8507-01	https://www.pattersonvet.com/ProductItem/078447425#productSpecificationsTab	Surgery (general use)
Buprenorphine	PAR Pharmaceutical	42023-179-05	https://www.parpharm.com/products/sterile/buprenorphine-hcl-injection/	Surgery (general use)
Proparacaine HCL Ophthalmic	Medline Industries	24208-730-06	https://www.medline.com/product/Proparacaine-HCL-Ophthalmic/Ophthalmic-Medications/Z05-PF87216?qu_4	Topical eye analgesic (retro-orbital injections)
**Implantable components**				
304 stainless steel	McMaster Carr	8983K114, 8983K113, 8983K111, 3254K322	https://www.mcmaster.com/products/304-stainless-steel/multipurpose-304-stainless-steel-6/ https://www.mcmaster.com/3254k322/	Implant
316 stainless steel	Proto Labs	316L CL 20ES	https://www.protolabs.com/services/3d-printing/direct-metal-laser-sintering/stainless-steel/	Implant
Laser-cutting service	Laser Alliance LLC	Milpitas, CA	https://laseralliance.com/	Implant
BioMed Clear	Form Labs	RS-F2-BMCL-01	https://formlabs.com/store/materials/biomed-clear-resin/	Implant
Surgical Guide	Form Labs	RS-F2-SGAM-01	https://formlabs.com/store/materials/surgical-guide-resin/	Implant
Grinding Wheel	WEN	4276 2.1-Amp 6-Inch	https://wenproducts.com/products/wen-bg4276-2-1-amp-6-inch-single-speed-bench-grinder-with-flexible-work-light	Implant
3D printed covers	Stratasys	uPrint ABS		Implant Cover
3D printed covers	Makerbot	Nylon 12 Carbon Fiber [375-0061A] or ABS-R [375-0071A]		Implant cover
Neodymium magnets	MacMaster Carr	5862K141	https://www.mcmaster.com/5862K141/	Implant Cover
Light Cure Adhesive LOCTITE 4311	Henkel	HL1401789	https://www.henkel-adhesives.com/us/en/product/uv-curing-adhesives/loctite_43110.html	Implant Cover
**Surgery Station Equipment**				
Compact Flexure Plate Clamp, 1/4"-20 Tap	Thorlabs	PC2	https://www.thorlabs.com/thorproduct.cfm?partnumber=PC2#ad-image-0	Clamps (surgery station)
Ø1/2" Optical Post, 1/4"-20 Tap, L = 6"	Thorlabs	TR6	https://www.thorlabs.com/thorproduct.cfm?partnumber=TR6	Posts (surgery station)
1/2" XYZ Translation Stage with Standard Micrometers, 1/4"-20 Taps	Thorlabs	PT3	https://www.thorlabs.com/thorproduct.cfm?partnumber=PT3#ad-image-0	XYZ micromanipulators (surgery station)
Aluminum Breadboard, 6" x 18" x 1/2", 1/4"-20 Taps	Thorlabs	MB618	https://www.thorlabs.com/thorproduct.cfm?partnumber=MB618	Base (surgery station)
Aluminum Breadboard, 4" x 12" x 1/2", 1/4"-20 Taps	Thorlabs	MB412	https://www.thorlabs.com/thorproduct.cfm?partnumber=MB412	Arm rests (surgery station)
Surgical Table (60'' x 30'')	McMaster-Carr		•https://www.mcmaster.com/4380T12/ •https://www.hubert.com/product/PJB.ST6-3060SBK/John-Boos-16GA-60X30-FLAT-WADJ-SS-BRC-KD	Surgery station
Homeothermic system	FHC	40-90-8D; 40-90-5D-02;	https://stoeltingco.com/Neuroscience/Homeothermic-Blanket-System~9905	Heating system (Surgery Station and acute imaging)
RightTemp® Jr. Heating Pad	Kent Scientific	RT-JR-15	https://www.kentscientific.com/products/righttemp-jr/	Surgery (general use)
Posi-Vac nosecone	Patterson Scientific	78914730	https://www.pattersonscientific.com/pos	Surgery (general use)

bioRxiv preprint doi: https://doi.org/10.1101/2023.05.22.541477; this version posted May 24, 2023. The copyright holder for this preprint (which was not certified by peer review) is the author/funder, who has granted bioRxiv a license to display the preprint in perpetuity. It is made available under aCC-BY 4.0 International license.

Item	Vendor	Product No.	Website	Experiment/Note
			i-vac-nonrebreathing-system/	
Isoflurane vaporizer system	VetEquip	911103	http://www.vetequip.com/item.asp?cat=3&catalogID=911103-6	Surgery (general use) and acute imaging)
EVAC 2 Waste Anesthetic Gas Evacuation System	Patterson Scientific	078918181 (Charcoal filter 78909457)	https://www.pattersonscientific.com/evac-2-evacuation-vacuum-attenuation/	Surgery (general use and acute imaging
Pump 11 Elite Nanomite Infusion/Withdrawal Programmable Syringe Pump	Harvard Apparatus	70-4507	https://www.harvardapparatus.com/remote-infuse-withdraw-pump-11-elite-nanomite-programmable-syringe-pump.html	Surgery (intraspinal injections)
Camera	The Imaging Source	DFK 33UX183c, IC Capture 2.5	https://www.theimagingsource.com/en-us/product/industrial/33u/dfk33ux183/	Window optical clarity validation
3D printed nose cone holder	Custom			Surgery (general use)
3D printed mouse surgery table/platform	Custom	Modified design from (Farrar et al., 2012).		Surgery (general use)
**Surgical reagents & tools**				
SURGIFOAM® Absorbable Gelatin Sponge	Ethicon	1972	https://www.ethicon.com/na/epc/code/1972?	•Surgery (general use, hemostatic)<br>•Similar to Gelfoam
Micro-Adson Forceps	Fine Science Tools	11018-12	https://www.finescience.com/en-US/Products/Forceps-Hemostats/Standard-Forceps/Micro-Adson-Forceps/11018-12	Surgery (general use)
Graefe Forceps	Fine Science Tools	11051-10	https://www.finescience.com/en-US/Products/Forceps-Hemostats/Standard-Forceps/Graefe-Forceps/11051-10	Surgery (general use)
Dumont #2 - Laminectomy Forceps	Fine Science Tools	11223-20	https://www.finescience.com/en-US/Products/Forceps-Hemostats/Dumont-Forceps/Dumont-2-Laminectomy-Forceps/11223-20	Surgery (general use)
Dumont #5 - Fine Forceps	Fine Science Tools	11254-20	https://www.finescience.com/en-US/Products/Forceps-Hemostats/Dumont-Forceps/Dumont-5-Fine-Forceps/11254-20	Laminectomy
Dumont #3c Forceps	Fine Science Tools	11231-20	https://www.finescience.com/en-US/Products/Forceps-Hemostats/Dumont-Forceps/Dumont-3c-Forceps/11231-20	Spared nerve injury
Fine Scissors - Sharp	Fine Science Tools	14060-10	https://www.finescience.com/en-US/Products/Scissors/Standard-Scissors/Fine-Scissors-Sharp/14060-10	Implant
Spring Scissors - 8mm Cutting Edge	Fine Science Tools	15023-10	https://www.finescience.com/en-US/Products/Scissors/Spring-Scissors/Spring-Scissors-(1)/15023-10-(1)	Implant
Spring Scissors - Angled	Fine Science Tools	15010-09	https://www.finescience.com/en-US/Products/Scissors/Spring-Scissors/Spring-Scissors-Angled/Spring-Scissor-Angled-2mm	Laminectomy
Tissue Separating Scissors	Fine Science Tools	14072-10	https://www.finescience.com/en-US/Products/Scissors/Standard-Scissors/Tissue-Separating-Scissors/14072-10	Spared nerve injury
Student Vannas Spring Scissors	Fine Science Tools	91500-09	https://www.finescience.com/en-US/Products/Scissors/Spring-Scissors/Student-Vannas-Spring-Scissors/91500-09-(1)	Spared nerve injury
Personnet Mini Retractor	Roboz	RS-6504	http://shopping.roboz.com/micro-scissors-micro-forceps-groups/Spreading/RS-6504-Personnet-Mini-Retractor-1-3X3-4mm-Sharp-Prongs	Implant (optional step)
Absorbable suture	Patterson Dental	PGA 6-0. #090-1660	https://www.pattersondental.com/Supplies/ProductFamilyDetails/PIF_537732	Implant
8-0 silk sutures	S&T	03192		Spared nerve injury
6-0 silk sutures	Henry Schein	101-2636	https://www.henryschein.com/us-en/Shopping/ProductDetails.aspx?productid=1012636&CatalogName=DENTAL	Spared nerve injury

bioRxiv preprint doi: https://doi.org/10.1101/2023.05.22.541477; this version posted May 24, 2023. The copyright holder for this preprint (which was not certified by peer review) is the author/funder, who has granted bioRxiv a license to display the preprint in perpetuity. It is made available under aCC-BY 4.0 International license.

Item	Vendor	Product No.	Website	Experiment/Note
Delicate bone scraper	Fine Science Tools	10075-16	https://www.finescience.com/en-US/Products/Bone-Instruments/Bone-Chisels/Delicate-Bone-Scraper/10075-16	Implant
Titanium Micro Spatula	Fine Science Tools	10167-11	https://www.finescience.com/en-US/Products/Spatulae,-Spoons-Curettes/Spatulae/Titanium-Micro-Spatula/10167-11	Implant
Micro drill	Foredom	K.1070	https://www.foredom.net/product/k-1070-high-speed-rotary-micromotor-kit-2-35mm-3-32-or-1-8-collet/	Laminectomy
Acu-Needle 33G – 1/2” (Needles)	Accuderm inc.	NP335	https://www.fishersci.com/shop/products/NC1145832/NC1145832	Implant
Universal Dentin Activator Gel (10% Citric acid, 3% Ferric chloride)	Parkell	S394	https://www.parkell.com/c-b-metabond_7	Implant (optional step)
3 mm coverslip (#0)	Warner Instruments	640726	https://www.warneronline.com/coverslip-coverglass	Window
1-mm borosilicate glass capillaries	WPI	1B100F-4	https://www.wpiinc.com/var-1953-standard-glass-capillaries	Intraspinal injection
PUL-100 puller	WPI	P-100		Intraspinal injection
Hamilton syringe	Hamilton	7653-01	https://www.hamiltoncompany.com/laboratory-products/syringes/7653-01	Intraspinal injection
Crescent blade - Micro Knife	Fine Science Tools	10317-14	https://www.finescience.com/en-US/Products/Scalpels-Blades/Micro-Knives/Micro-Knives-Plastic-Handle/10317-14	Laminectomy
Electronic Super Knips®	Knipex	7861125	https://www.knipex.com/products/electronics-pliers/electronic-super-knips/electronic-super-knips/7861125	Implant
**Adhesives**				
Vetbond	3M	1469SB	https://www.3m.com/3M/en_US/p/d/v000058033/	Implant
Super Glue	Loctite	1365882	https://www.loctiteproducts.com/en/products/fix/super-glue/loctite_super_glueliquidprofessional.html	Implant
Low Toxicity Silicone Adhesive	WPI	KWIK-SIL	https://www.wpiinc.com/kwik-sil-low-toxicity-silicone-adhesive?	Implant, Laminectomy, Window
C&B Metabond® Quick Adhesive Cement System PMMA (includes universal gel dentin activator)	Parkell	S380	https://www.parkell.com/c-b-metabond_3	Implant, Window
Norland Optical Adhesive NOA 81	Edmund Optics	36-428	https://www.edmundoptics.com/p/1-oz-application-bottle-of-noa-81/4179/	Window (alternative to cement)
**General use**				
Patterson® Disposable Micro Applicator – 0.5 mm	Patterson Dental Supply	070826198	https://www.pattersondental.com/Supplies/ItemDetail/070826198	Surgery (general use)
Weck-Cel® Cellulose Eye Spears	BVI Medical	0008680	https://www.bvimedical.com/products/fluid-control/	Surgery (general use)
UV flashlight	LIGHTFE (Amazon)	FL 365NM	https://www.amazon.com/gp/product/B07BC8L581/ref=ppx_yo_dt_b_search_asin_title?ie=UTF8&psc=1	Window (alternative to cement)
Nair™ hair removal cream	Nair	22600223191	http://www.naircare.com/en/women/products/nair-lotion-with-baby-oil	Implant
Wahl Professional Shaver	Wahl Clipper Corp	8685	https://www.wahlpro.com/shop/classic-peanut-08685	Surgery (spared nerve injury)
Press’n Seal®	Glad	Press’n Seal®	https://www.glad.com/food-storage/plastic-wrap/press-n-seal-home-collection	Surgery (general use)
SYSTANE® NIGHTTIME Eye Ointment	Alcon	02444062	https://systane.myalcon.com/eye-care/systane/products/systane-nighttime/	Surgery (general use)
Povidone Iodine Prep Solution, 4 oz.	Medline	MDS093944H	https://www.medline.com/sku/item/MDPMDS093944H?	Implant
**Synthetics**				

bioRxiv preprint doi: https://doi.org/10.1101/2023.05.22.541477; this version posted May 24, 2023. The copyright holder for this preprint (which was not certified by peer review) is the author/funder, who has granted bioRxiv a license to display the preprint in perpetuity. It is made available under aCC-BY 4.0 International license.

Item	Vendor	Product No.	Website	Experiment/Note
Teflon AF 2400 membrane	VICI Metronics	AF-050-025-025 (25mm x 25mm x 50µm)	https://www.vicimetronics.com/collections/random-technologies-teflon-af-products/products/teflon-af-films-1	Window
GORE® PRECLUDE® Pericardial Membrane	Gore Medical (acquired through Dotmed.com or other resale vendor)	1PCM101	https://www.goremedical.com/products/precludepericardial	Laminectomy
**Viruses**				
AAV-PHP.eB-CAG-nls-GFP	Addgene	104061-PHPeB	https://www.addgene.org/104061/	Retro-orbital injections
AAV-PHP.S-CAG-tdTomato	Addgene	59462-PHP.S	https://www.addgene.org/59462/	Retro-orbital injections
AAV2retro-hSyn-Cre	Addgene	105553-AVVrg	https://www.addgene.org/105553/	Intraspinal injection
**In vivo imaging setup**				
VIVO Multiphoton	3i		https://www.intelligent-imaging.com/vivo-multiphoton	
Mug Warmer Cooler	Yeosen	PH-F3		Sensory stimuli (awake)
Hot plate	Fisher Scientific	HP88854200	https://medexsupply.com/fisher-scientific-isotemp-ceramic-hotplate-4-25-x-4-25-120v-60hz/	Sensory stimuli (awake)
Glass Syringes	Synthware™	S371202	https://www.fishersci.com/shop/products/graduated-borosilicate-glass-syringes/31502318	Sensory stimuli (awake)
Brush	NicPro	MG015, Flat 1 and Round 1	https://nicpro.com/products/nicpro-micro-detail-paint-brush-set-15-tiny-professional-miniature-fine-detail-brushes-detailing-paint-kit-for-spray-watercolor-oil-acrylic-craft-models-painting	Sensory stimuli (awake)
Forceps (Pinch)	AVEN	18434	https://www.aventools.com/aven-8-forceps-wstraight-serrated-tips	Sensory stimuli (awake)
Force sensitive resistor	Adafruit	166	https://www.adafruit.com/product/166	Sensory stimuli (awake)
Compressed gas duster	Dust-off	8541677532, 8541677551		Air puff stimulus
Piezo Buzzer	Intervox	BRP3018L-12-C	https://us.rs-online.com/product/icc-intervox/brp3018l-12-c/70115792/	Sound Stimulus
Low-Profile Anesthesia Mask	Kent Scientific	SOMNO-0801	https://www.kentscientific.com/products/low-profile-anesthesia-masks-for-somnosuite/	Acute imaging
Ultrasound gel	Parker Labs	638632490755 (01-34)	https://www.parkerlabs.com/products/aquasonic-100-ultrasound-transmission-gel/	Imaging
Infrared acrylic	ePlastics	ACRY31430.125PM11.555x11.850	https://www.eplastics.com/ACRY31430-125PM11-555X11-850	Mouse blinders and circular running wheel
Infrared 850 nm IR LED Strip Light	Waveform Lighting	7031.85	https://store.waveformlighting.com/products/infrared-850-nm-ir-led-strip-light	NIR imaging
Infrared Night Vision Illuminator	LIYUDL	B071KPSGCT	https://www.amazon.com/LIYUDL-48-LED-Infrared-Illuminator-Security/dp/B071KPSGCT	NIR imaging
JC Infrared Illuminator	Shenzhen Jing Cheng Digital Surveillance Co,	IRINB04L	https://www.amazon.com/Infrared-Illuminator-Power-Vision-Camera/dp/B01D73XM24	NIR imaging
Red LED Flexible Strip Ribbon Light	ALITOVE	AL5RWPBK12V	https://www.amazon.com/ALITOVE-Flexible-Waterproof-Commercial-Lighting/dp/B07MXXDWDM?th=1	LED
FilmGrade™ Flicker-Free LED Dimmer	Waveform Lighting	3081	https://store.waveformlighting.com/products/filmgrade-flicker-free-dimmer-for-led-strip?src=pse	LED dimmer
Goniometer	Thorlabs	GNL20	https://www.thorlabs.com/thorproduct.cfm?partnumber=GNL20	Setup
Jack	Thorlabs	L490	https://www.thorlabs.com/thorproduct.cfm?partnumber=L490	Setup
Mounting bracket	Thorlabs	C1515	https://www.thorlabs.com/thorproduct.cfm?partnumber=C1515	Setup
Aluminum perforated sheet	McMaster Carr	92725T51	https://www.mcmaster.com/92725T51/	Setup (circular running wheel)

bioRxiv preprint doi: https://doi.org/10.1101/2023.05.22.541477; this version posted May 24, 2023. The copyright holder for this preprint (which was not certified by peer review) is the author/funder, who has granted bioRxiv a license to display the preprint in perpetuity. It is made available under aCC-BY 4.0 International license.

Item	Vendor	Product No.	Website	Experiment/Note
Rotary encoder	Signswise	LN11-ERGA	https://www.amazon.com/Signswise-Incremental-Optical-Encoder-Quadrature/dp/B00Y9KDDCY?th=1	
3D printed parts	Custom			Connects running wheel to the rotary encoder
3D printed part	Custom			Attaches rotary encoder to Thorlabs posts.
245mm Square Dishes	Corning	07-200-600	https://www.fishersci.com/shop/products/corning-245mm-square-bioassay-dishes-3/07200600	Setup (plastic cover)
Saleae Logic 8	Saleae	SAL-00111	https://usd.saleae.com/products/saleae-logic-8	Setup (logic analyzer)
Decibel Meter	Tadeto	SL720	https://www.amazon.com/Tadeto-Portable-Backlight-Weighted-Factories/dp/B09T37812W?th=1	Sound meter
Compact Power and Energy Meter Console	Thorlabs	PM100D	https://www.thorlabs.com/thorproduct.cfm?partnumber=PM100D	Power output for imaging
Standard Photodiode Power Sensor	Thorlabs	S120C	https://www.thorlabs.com/thorproduct.cfm?partnumber=S120C	LED power output for one-photon imaging
Standard Photodiode Power Sensor	Thorlabs	S121C	https://www.thorlabs.com/thorproduct.cfm?partnumber=S121C	Laser power output for two-photon imaging
Hirose cable	The Imaging Source	CA-x2-HIR-OE/1.5		Sync cameras with imaging
Fluorescent microspheres	Invitrogen	F8765		
**Cameras for one-photon imaging**				
CoolSNAP EZ	Teledyne Photometrics		https://www.photometrics.com/wp-content/uploads/2019/10/CoolSNAP-EZ-Datasheet.pdf	
Kinetix	Teledyne Photometrics		https://www.photometrics.com/products/kinetix-family/kinetix	
Fusion BT	Hamamatsu Photonics K.K.	C15440-20UP	https://www.hamamatsu.com/eu/en/product/cameras/cmos-cameras/C15440-20UP.html	
Prime BSI	Teledyne Photometrics		https://www.photometrics.com/products/prime-family/primebsi	
pco.edge 4.2 bi	Excelitas PCO GmbH		https://www.pco.de/scientific-cameras/pcoedge-42/	
Axiocam 712	Zeiss			
**Behavior room**				
Lamp	JOOFO	Clipper0722-54	https://www.amazon.com/JOOFO-Torchiere-Temperatures-Lamps-Tall-Office%EF%BC%88Black%EF%BC%89/dp/B07WFC14VR	Behavior (general use)
Lamp	Barrina	INWT504005650Fc	https://www.amazon.com/Barrina-Dimmable-Adjustable-3000K-5500K-Flexible/dp/B078S57SG4	Behavior (general use)
Digital Lux meter	JRLGD	LX1010B	https://drmeter.com/products/digital-lux-meter-digital-illuminance-light-meter-lx1010b-dr-meter	Behavior (general use)
Camera	The Imaging Source	DMK 21AU04, DFK 42BUC03	•https://www.theimagingsource.com/en-us/product/industrial/2u/dfk42buc03/ •https://dl-gui.theimagingsource.com/en_US/316abbc0-5e27-575c-b0ef-ded415604df4/	Behavior (general use)
Touch Test™ Sensory Probes, Stoelting	Stoelting	58011	https://us.vwr.com/store/product/23125281/touch-testtm-sensory-probes-stoelting	von Frey filaments
Rotarod	Ugo Basile	7650, 47650	https://ugobasile.com/products/categories/motory-coordination/rotarod-for-mice-and-rats	Behavior (Rotarod test)
Arena 2-ft diameter, white	TAP Plastics	HDPE		Behavior (Open field)
15.5–16’’ tall white walls	Mr. Plastics	WHT POLYSTYRENE		Behavior (Open field)
